# Influence of task decision autonomy on physical ergonomics and robot performances in an industrial human–robot collaboration scenario

**DOI:** 10.3389/frobt.2022.943261

**Published:** 2022-09-27

**Authors:** Matteo Pantano, Qiaoyue Yang, Adrian Blumberg, Raven Reisch, Tobias Hauser, Benjamin Lutz, Daniel Regulin, Tobias Kamps, Konstantinos Traganos, Dongheui Lee

**Affiliations:** ^1^ Functional Materials and Manufacturing Processes, Technology Department, Siemens Aktiengesellschaft, Munich, Germany; ^2^ Human-Centered Assistive Robotics (HCR), Department of Electrical and Computer Engineering, Technical University of Munich (TUM), Munich, Germany; ^3^ Industrial Engineering and Innovation Sciences, School of Industrial Engineering, Eindhoven University of Technology, Eindhoven, Netherlands; ^4^ Autonomous Systems, Technische Universität Wien, Vienna, Austria; ^5^ Institute of Robotics and Mechatronics, German Aerospace Center (DLR), Wessling, Germany

**Keywords:** physical ergonomics, autonomy, human-robot collaboration (HRC), RULA, musculoskeletal disorders

## Abstract

Adoption of human–robot collaboration is hindered by barriers in collaborative task design. A new approach for solving these problems is to empower operators in the design of their tasks. However, how this approach may affect user welfare or performance in industrial scenarios has not yet been studied. Therefore, in this research, the results of an experiment designed to identify the influences of the operator’s self-designed task on physical ergonomics and task performance are presented. At first, a collaborative framework able to accept operator task definition *via* parts’ locations and monitor the operator’s posture is presented. Second, the framework is used to tailor a collaborative experience favoring decision autonomy using the SHOP4CF architecture. Finally, the framework is used to investigate how this personalization influences collaboration through a user study with untrained personnel on physical ergonomics. The results from this study are twofold. On one hand, a high degree of decision autonomy was felt by the operators when they were allowed to allocate the parts. On the other hand, high decision autonomy was not found to vary task efficiency nor the MSD risk level. Therefore, this study emphasizes that allowing operators to choose the position of the parts may help task acceptance and does not vary operators’ physical ergonomics or task efficiency. Unfortunately, the test was limited to 16 participants and the measured risk level was medium. Therefore, this study also stresses that operators should be allowed to choose their own work parameters, but some guidelines should be followed to further reduce MSD risk levels.

## 1 Introduction

Small-batch manufacturing is becoming increasingly important for the competitive advantage of European factories, especially within small and medium enterprises (SMEs) (Bayha et al., 2020). Technologies proposed in Industry 4.0 (I4.0) can enable economically feasible small-batch manufacturing (Rüßmann et al., 2015). One of the promising technologies within I4.0 is human–robot collaboration (HRC). In HRC, humans are foreseen to collaborate with robots in a shared workspace to achieve higher flexibility and throughput. However, their introduction is still hampered by safety, interfaces, and design (Villani et al., 2018). To address these problems, including the human operator in the design *via* human-driven design paradigms can be beneficial (Kaasinen et al., 2019). In such scenarios, the design paradigms known as human-centered design (HCD) (Deutschen Instituts für Normung, 2020) and value-sensitive design (VSD) (Friedman, 1996) have helped to improve the usability of robotic systems for novice operators (Coronado et al., 2021; Eiband et al., 2022), reduce workload by using custom designed interfaces (Pantano et al., 2020), or improve acceptance by changing the appearance of humanoid robots (Kahn et al., 2007). However, to achieve these results, design must aim to establish means of communication that enable humans to build good mental models of the application (Rook, 2013; Sofge, 2013; Hoff and Bashir, 2015; Teo et al., 2018; Demir et al., 2019; Kolbeinsson et al., 2019; Shahrdar et al., 2019). One recent example proving the benefit of good mental models can be seen in the work of (Tausch and Kluge, 2020). In this work, the authors found that greater satisfaction in human–robot interaction can be achieved if operators design their own sequence of tasks. However, no investigation in industrial scenarios was performed in the study. Therefore, there is a need for further research in this sector. By looking at the adoption of HRC in industry, the applications which could benefit the most from those design suggestions are the ones known as cooperation and collaboration (Weiss et al., 2021). In these modes, teammates (i.e., human and robot) perform tasks in a shared workspace on different components or on the same components (Bauer et al., 2016). If these modes are successfully implemented through good mental models, several benefits can be achieved. One of those is the improvement of the operators’ physical ergonomics (Gualtieri et al., 2021). Therefore, the following section describes how mental models were implemented when the physical ergonomics of operators had to be taken into account in an HRC application.

To consider the physical ergonomics of operators in HRC, an assessment must be carried out. In the literature, two assessment methodologies are available: simulation *via* computer-aided engineering (CAE) and digital human models (DHMs) (Sanchez-Lite et al., 2013; Baizid et al., 2016; Mgbemena et al., 2020) or *in situ* process surveys (Lidstone et al., 2021). In the case of CAE simulations, the digital workcell, the digital task workflow, and the DHM must be available (Gläser et al., 2016). This is often the case when planning new automotive production lines (Ruiz Castro et al., 2017; Zhu et al., 2019). Therefore, several commercial tools are available in the market such as IPS IMMA™^1^ or Siemens Tecnomatix™^2^. However, given the complexity of such commercial systems, they cannot be always applied in the context of SMEs, and the level of freedom for the operator is rather limited (Ballestar et al., 2020). In the case of *in situ* measurements, experts are requested to monitor the task and provide evaluations. This is often performed through the classification of operator postures through observations (Namwongsa et al., 2018). However, not surprisingly, this latter method can be subject to errors due to the observational source (Diego-Mas et al., 2017). Despite that, the *in situ* measurement is more flexible and does not require intensive digitization like the CAE method. Hence, the *in situ* approach, with proper technologies for reducing errors, has been widely adopted for estimating and improving physical ergonomics in HRC. Rahal et al., 2020 proposed a haptic control based on an inverse kinematics (IK) of the human arm to derive user comfort and thus change the control strategy. This strategy leads to lower muscular loads of operators by considering a physical ergonomics measurement in the algorithm. Shafti et al., 2019 presented a robot-assisted interaction that improves the operator armload by controlling the robot arm positioning in accordance with muscular and physical ergonomics measurements. Their approach tunes the robot response according to the physical ergonomics obtained through computer vision (CV). Makrini et al., 2019 suggested that human and robot task allocation based on physical ergonomics can improve the overall working conditions of the operators. Their result was based on a visual module for estimating the operator position, but it was influenced by a human posture tracking algorithm in sub-optimal operation. A similar approach, but with an improved visual algorithm based on OpenPose (Cao et al., 2017), can be used to derive compliant robot motions that follow postures of different operators, thus reducing the operator joint torque overloading (Kim et al., 2019).

The proposed *in situ* methodologies are mainly presenting robot control algorithms which adapt to the human posture to improve the physical ergonomics in the hand-over task. Therefore, individuals cannot explicitly program the robot behavior but should trust the robot control algorithm to choose the most comfortable position. Although beneficial to the user physical ergonomics, this could lead to rising feelings of uncertainty which can influence team dynamics (Friedman et al., 2000; Kolbeinsson et al., 2019; Tausch and Kluge, 2020). Therefore, studies investigating the effect of individuals’ explicit decisions on task design in industrial scenarios are missing. Thus, this work investigates if mental models based on a self-designed task in an industrial scenario influence the operator in terms of physical ergonomics and task performance. To study these influences, we measure the level of physical ergonomics in two experiments with different levels of task autonomy and we formulate the following research hypotheses.

Hypothesis 1 (*H1*): The worker, through the ability to explicitly define the location of objects to be manipulated, has a high task decision autonomy.

Hypothesis 2 (*H2*): When the worker can explicitly define the location of the objects and perceives more autonomy, the physical ergonomics of the operator is better.

To test these research hypotheses, the work in this article is structured as follows. In Section 2, the use case and the envisioned novel control method based on an adaptive control architecture that leaves the decision on where to place the parts to the operator are described. In Section 3, the results of a user test with untrained personnel on physical ergonomics data are presented. In Section 4, the results considering the research hypotheses and possible factors influencing the outcomes are discussed. Finally, in Section 5, the conclusions along with future research directions are given.

## 2 Materials and methods

This section discusses the materials and methods used to test the research hypotheses through experiments. First, the task and the interaction envisioned for the experiments are described in Section 2.1. Second, the methods to estimate the physical ergonomics are described in Section 2.2. Third, Section 2.3 and Section 2.4 describe how the user could specify the location of the objects and operate safely with the robot. Finally, Section 2.5 describes the experiment and the procedure for gathering data from the user study.

### 2.1 Task description and envisioned interaction

To efficiently produce parts that require sintering processes, batch production is applied. Therefore, stacks composed of several insulating layers and parts must be prepared before thermal sintering (Verlee et al., 2012). However, due to the fragility of some parts, this process is often performed manually, resulting in production errors and strain for human operators (Murrell, 1961; Hansen et al., 2003).

To integrate the self-designed task while reducing operator effort, a VSD-based approach was used to ensure a holistic solution. Therefore, users were observed during the manual task, and two conclusions were drawn. On the one hand, the stakeholders are the human operators involved in the stacking process. On the other, the users are moving the pieces as they please as long as they have their own way of performing the task. Therefore, the value of autonomy was highlighted as the most important thing because of the operators’ autonomy in the task. Moreover, the value of human well being was identified as second most important considering that the task can be strenuous for operators.

**FIGURE 1:**
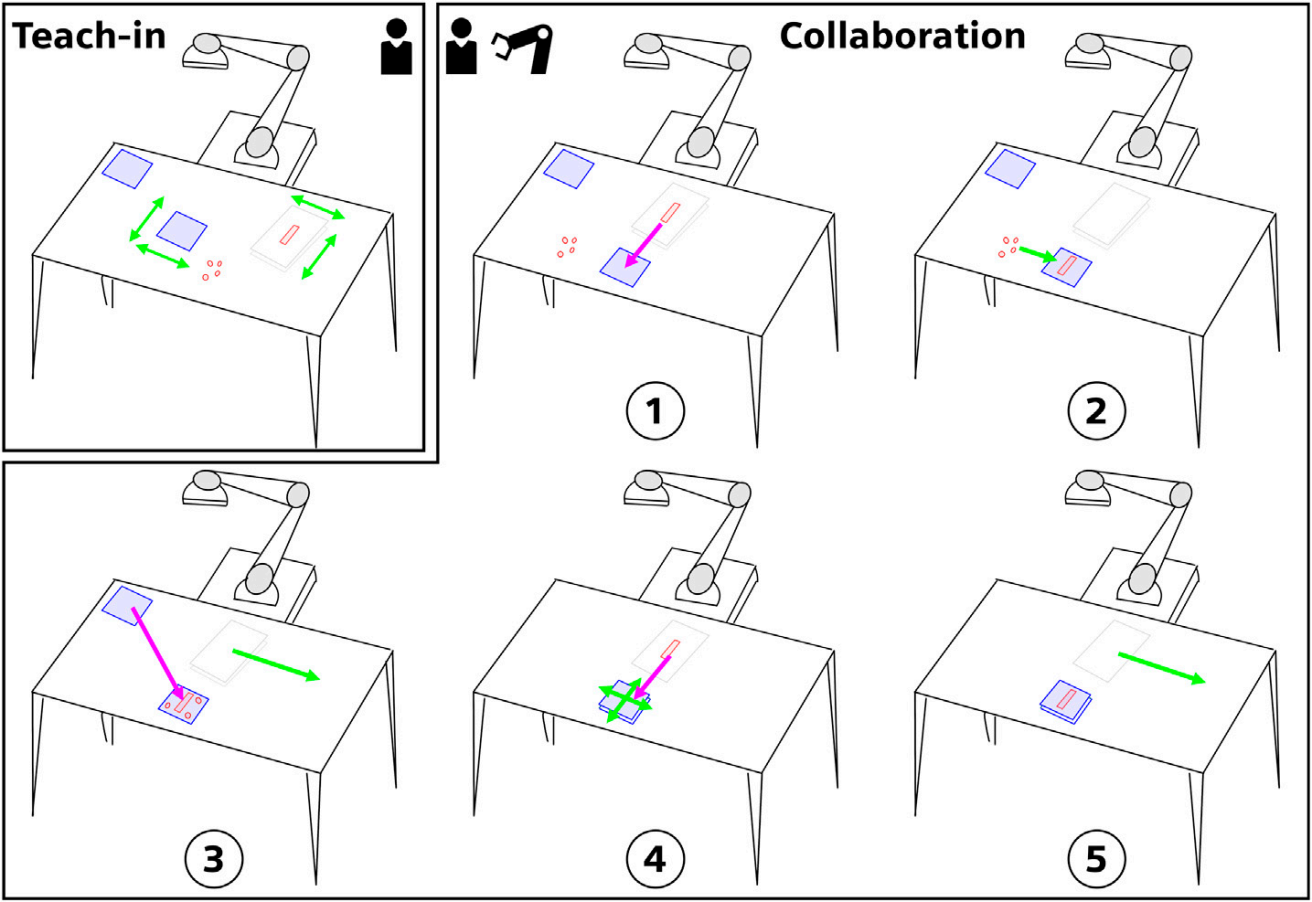
Graphical representation of the envisioned interaction. The task is composed of a teach-in phase and a collaboration phase. At first, the user decides where the parts should be placed. Afterward, the collaboration unfolds with 5 sub-steps where the robot and the user have to share some tasks. Step ➀ the robot moves the part to the insulating layer. Step ➁ the user places the distancers on the insulating layer. Step ➂ the robot moves another insulating layer while the user moves away the paper sheet. Step ➃ the robot moves the part while the user adjusts the insulating layer stack. Step ➄ the user moves away the paper sheet. The actions marked with light green are taken over by the human operator. The actions marked in pink are taken over by the robot.

Considering these values, and the limitations found in SMEs where application of CAE modeling and robot offline programming like in (Baizid et al., 2016) is limited, the following twofold approach has been selected. A human collaboration approach was chosen to alleviate operators’ efforts and CV to identify where the robot should pick up parts and give operators the freedom to customize the task. Therefore, the envisioned interaction was composed of two parts. First, a teach-in phase where the user could exert autonomy by placing parts at preferred location. Second, the collaboration for fulfilling the task of stack creation. The final task workflow is shown in Figure 1.

### 2.2 Posture evaluation

To investigate the influence of human decisions on posture, an evaluation method was necessary. As pointed out previously, an approach based solely on expert observation can carry errors. Therefore, an approach based on convolutional neural networks (CNNs) and CV was selected. The method was integrated *via* the pretrained limb detection algorithm OpenPose (Cao et al., 2017) and a calculation library for joint angles. These two tools were used to evaluate the operator’s distances to the workspace and the exposure to ergonomic risk factors related to musculoskeletal disorders (MSDs) following the Rapid Upper Limb Assessment (RULA) method (McAtamney and Nigel Corlett, 1993), due its better performance in calculating risks ranging from low to high (Yazdanirad et al., 2018).

**FIGURE 2:**
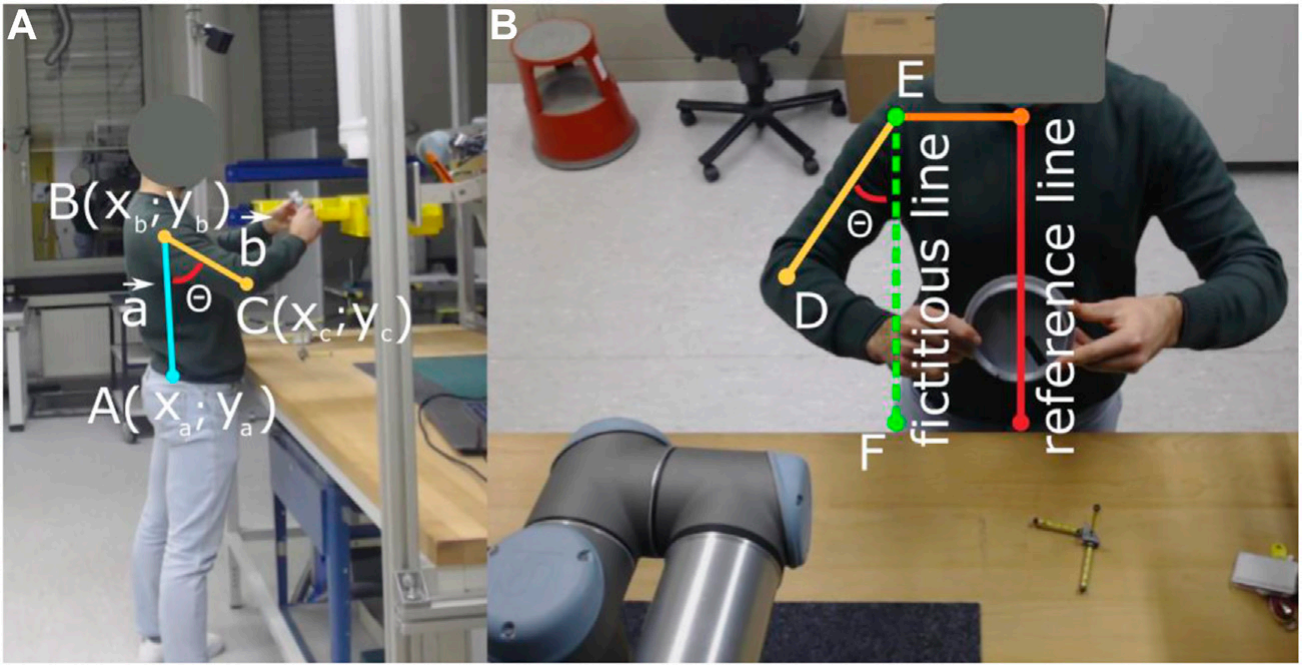
Example for the evaluation of the operator angles using front and side views. The view from the front is necessary to get information about the upper limbs, and the view from the side is necessary to get information about lower limbs and trunk position. The calculation of the angles is performed between adjacent limbs **(A)**. In case no adjacent limbs were present, a reference line had to be selected to create a fictitious line for the calculation **(B)**.

To implement the RULA calculation, a similar approach to the one used by (Makrini et al., 2019) was used. Therefore, the operators were monitored from two points of view *via* different cameras, one for recording information of the upper limbs (front) and one for the lower limbs (side). Afterward, the views were synchronized, and all relevant limb positions were extracted and evaluated as shown in Figure 2. More precisely, to calculate RULA relevant angles, the limb end points according to Table 1 were used and angles were calculated using Eq. 1 (nomenclature refers to labels in Figure 2; the numerator is the dot product of the two vectors representing two adjacent limbs and the denominator is the multiplication of the lengths of the two limb vectors).

$$\theta =\cos^{-1}\frac{\vec{a}\;\cdot \;\vec{b}}{\left||\vec{a}\left||\;|\right|\vec{b}|\right|}$$ (1)

**TABLE 1:** Key points used for the RULA evaluation using the side view. The first column specifies which RULA criteria is considered. The second column explains which key points from the OpenPose output were considered for the angle calculation. Finally, the last column identifies which reference line was taken to create the fictitious line in case the relevant key points were not adjacent.

RULA criteria	Relevant key points of limbs	Reference line
Upper arm position	Shoulder—elbow	Trunk
Shoulder raise	Neck—shoulder	Trunk
Lower arm position	Elbow—wrist	Trunk
Wrist position	Wrist—palm	Elbow—wrist
Neck position	Neck—ear	Trunk
Trunk position	Trunk	Perpendicular line to the ground

Finally, the physical ergonomics score was calculated according to the RULA criteria using lookup tables to convert the quantitative limb angle information into the ordinal data needed for RULA (this conversion assumed that a constant load under 0.5 kg and non-repetitive actions due to the task structure were present). More precisely, the angles calculated using Eq. 1 were used to give intermediate risk levels for all the different limbs (e.g., upper arm). Then, the intermediate risk levels were used to define target rows and columns in the RULA tables. Finally, the cell identified by the row and column gave the final MSD risk level. For the sake of clarity, an example is presented here. The example is composed of two steps following the RULA evaluation. On one side, there is an intermediate MSD risk level for the wrist and arm, and on the other side, there is an intermediate MSD risk level for the neck, trunk, and legs. First, if the upper arm had an angle between +20^∘^ and -20^∘^, the lower arm was bent for more than 100^∘^, and the wrist was parallel to the ground and without twisting, an intermediate limb risk level equivalent to 1, 2, 1, and 1 was obtained. Therefore, this yielded an intermediate risk level of 2 for the wrist arm. Second, if the neck and trunk were straight and the legs were supported, an intermediate limb risk level equivalent to 1, 1, and 1 was obtained. Therefore, this yielded an intermediate risk level of 3 for the neck, trunk, and legs. Finally, by combining the results of the first and second steps, the final MSD risk level was obtained by looking at the second row and third column of the RULA evaluation table^3^, which, in this case, results in a final MSD risk level of 3.

In addition to the main RULA assessment, the posture assessment was supplemented with a measurement of the distance between the robot’s work area and the operator to get an overview of work area utilization. Therefore, another CV method was applied. The pipeline was as follows. Initially, the output from OpenPose was taken, and the front view was selected to calculate the average operator distance using shoulder and hip positions with the $L^{2}\;norm$ as show in Eq. 2.

$$avgPos=\sqrt[{2}]{shoulderPose^{2}+hipPose^{2}\;}$$ (2)

Afterward, the boundary between the working areas (table edge) was identified. Finally, the distance between the boundary and the operator was calculated using the $L^{2}\;norm$ , and an average value was calculated using Eq. 3.

$$avgDist=\;\frac{\sum_{i=0}^{N}\sqrt[{2}]{boundaryPose_{i}^{2}+avgPose_{i}^{2}\;}}{N}$$ (3)

To perform these evaluations during the experiment, the information was processed using a script written in Python and run on a Linux computer that had access to camera video streams and robot status using the robot operating system (ROS) middleware. The source code for performing these evaluations has been made available^4^.

### 2.3 Adaptive robot control architecture

**FIGURE 3:**
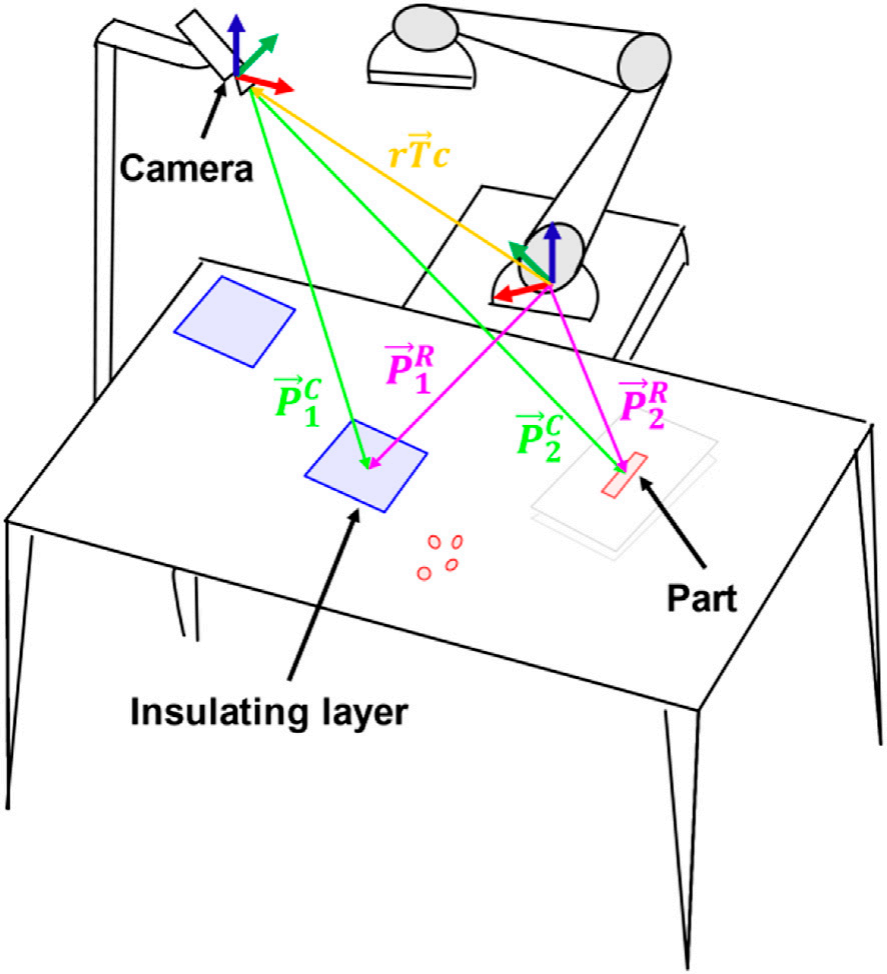
Coordinate transform problem. For the robot to be able to handle the parts, the transformations marked with ${\vec{P}}_{1}^{C},{\vec{P}}_{2}^{C}$ , ${\vec{P}}_{1}^{R},{\vec{P}}_{2}^{R}$ , and $r\vec{T}c$ are needed. The first two points ( ${\vec{P}}_{1}^{C},\;{\vec{P}}_{2}^{C}$ ), marked in green in the figure, define the position of the parts in the camera coordinate system, and they are obtained through a CNN algorithm. The second two ( ${\vec{P}}_{1}^{R},\;{\vec{P}}_{2}^{R}$ ), marked in pink in the figure, define the position of the parts in the robot coordinate system, and their position is necessary for the robot to handle the parts. To get this last information, the transformation between the robot and camera ( $r\vec{T}c$ ), marked in orange above, was obtained *via* the Perspective-n-Point algorithm (Lepetit et al., 2009).

To achieve a level of robot control which could be easily adapted to the requirements of the operator, a distributed cyber-physical system had to be conceived. The system consisted of four main parts: an application controller, a manipulator trajectory planner and executor, an object detector, and a low-level end-effector controller. The requirements for this structure were determined by the necessity of handling objects placed at user-defined locations. Therefore, capability for adaptation had to be considered. In this case, flexibility was integrated through a parametrizable solution of the robot IK *via* target coordinate frames. For the sake of clarity, the problem is shown in Figure 3. The pipeline to solve the IK started from the object detector, which initially analyzed the images and identified the different objects *via* an appropriately trained CNN (Redmon et al., 2015). Afterward, the identified objects’ pixel coordinates were transformed to the camera coordinate system by knowing the camera intrinsic parameters and assuming a pinhole camera model. This was achieved using Eq. 4 ( $u_{i},v_{i}$ are the pixel coordinates of the identified projected object, $c_{x},c_{y}$ are the coordinates of the principal point of the camera frame in the image center, and $f_{x},f_{y}$ are the focal lengths of the camera expressed in pixels).

$$P_{i}^{C}=\left[\begin{array}{cccc} \frac{\left(u_{i}-\;c_{x}\right)}{f_{x}} & \frac{\left(v_{i}-\;c_{y}\right)}{f_{y}} & 1 & 1 \end{array}\right],\forall i$$ (4)

Next, having the coordinates of the object in the camera coordinate system, the coordinates in the robot coordinate system were needed. To obtain those, the homogeneous transformation matrix between the camera and robot frame ( $r\vec{T}c$ in Figure 3) was calculated through the Perspective-n-Point algorithm (Lepetit et al., 2009) was obtained *via* a camera calibration routine^5^. The obtained transformation is expressed by Eq. 5 ( N is the number of singular values obtained from different camera poses, $\beta_{i}$ are initial coefficients, and $v_{i}$ are the right singular vectors).

$$r\vec{T}c=\;\left[\begin{array}{cccc} r_{11} & r_{12} & r_{13} & t_{1}\\ r_{21} & r_{22} & r_{23} & t_{2}\\ r_{31} & r_{32} & r_{33} & t_{3}\\ 0 & 0 & 0 & 1 \end{array}\right]=\;\sum_{i=1}^{N}\beta_{i}v_{i}$$ (5)

Finally, point-to-point trajectories were generated for reaching the target positions calculated through Eq. 6 by solving the robot’s IK problem ( s is the scaling factor dependent on the camera, and $x_{i}^{R},y_{i}^{R},\;z_{i}^{R}$ are the coordinates of the objects in the robot coordinate frame).

$$P_{i}^{R}=\left[\begin{array}{c} x_{i}^{R}\\ y_{i}^{R}\\ z_{i}^{R}\\ 1 \end{array}\right]=r\vec{T}c*P_{i}^{C}*s,\forall i$$ (6)

### 2.4 Cyber-physical system implementation

**FIGURE 4:**
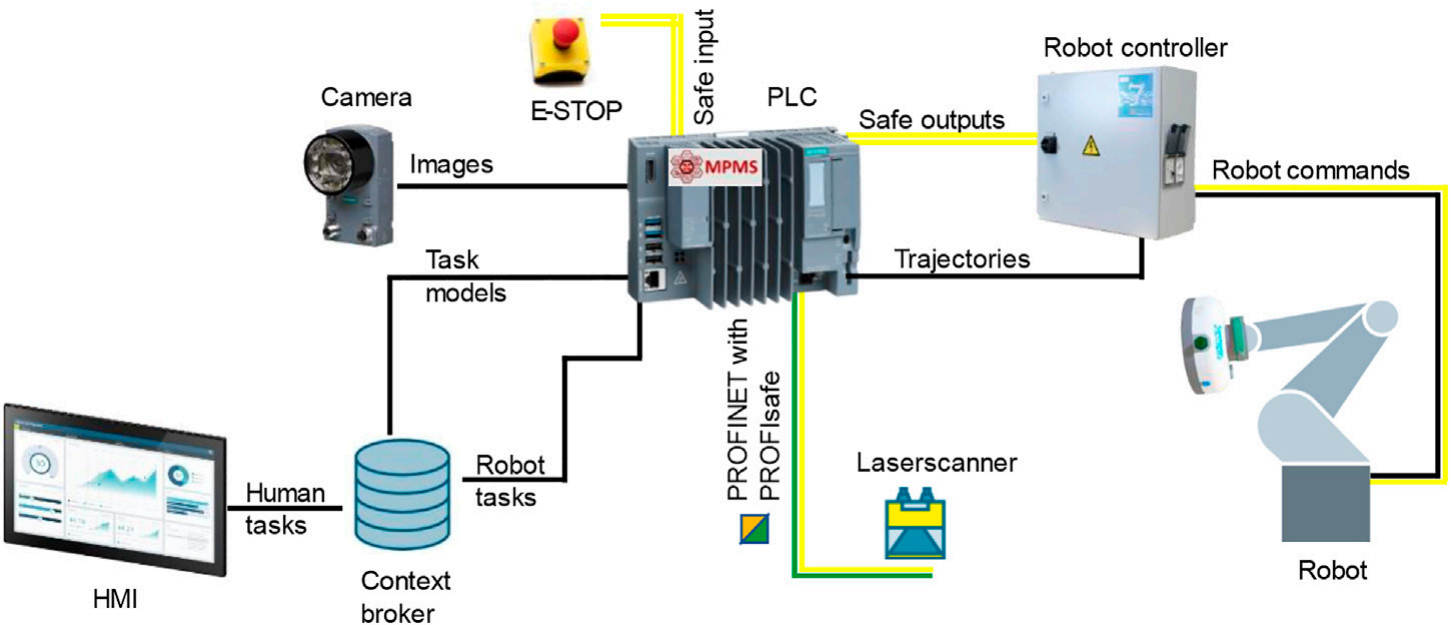
Network topology. The connections within the cyber-physical system allowed the implementation of speed and separation monitoring (SSM) and other risk reduction measures. The implemented hardware was Siemens^®^ S7-1500, Sick^®^ microScan3, Universal Robot^®^ UR10, Siemens^®^ Unified Control Panel, and an ethernet camera to get the images of the part displacement. Moreover, to control the different systems, the SHOP4CF architecture was adopted, employing the Manufacturing Process Management System (MPMS) component and the Task data model.

**FIGURE 5:**
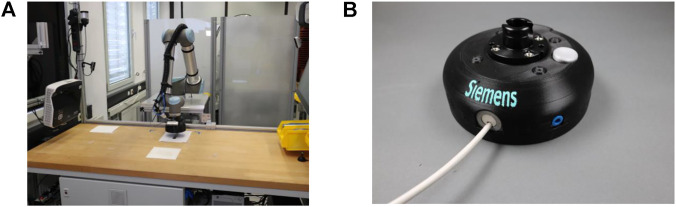
Implemented cyber-physical system and the gripper. The cyber-physical system is an ensemble of safety devices to guarantee safe cooperation and actuators for handling the parts of the use case. The robot is placed on one side of the table and the user had to stand in front of it to have a clear view of the robot motions **(A)**. Moreover, the laser scanner was placed in front of the table so that it could promptly measure the presence of operators in the workspace and drive the robot to a slower operating speed. The specially designed gripper tested for safe human–robot collaboration was made using 3D printing and implemented mechanical principles which allowed grasping of the components in the use case **(B)**.

To ensure a safe collaboration between the robot and the operator, the implemented cyber-physical system had to consider the current safety regulations. Hence, the ISO/TS 15066 (International Organization for Standardization, 2016) and the ISO 12100 (International Organization for Standardization, 2010) were followed. Therefore, the safety modality known as power and force limiting (PFL) was selected to reduce the risks identified by the hazard analysis conducted according to ISO 12100 (2010). For allowing the implementation of the modalities, a safe Programmable Logic Controller (PLC) Siemens^®^ S7-1500 together with a Sick^®^ microScan3 were used with a network topology as shown in Figure 4. Moreover, to consent to the integration of PFL, a robot with ISO10218-1 (International Organization for Standardization, 2011) certification had to be selected, in this case, a Universal Robot^®^ UR10 was used. Finally, to complete the integration of PFL, a custom gripper for the needs of the use case was developed and collision tests were performed to determine a safe operating speed as proposed by (Pantano et al., 2021). The final workcell with the implemented features is shown in Figure 5.

**FIGURE 6:**
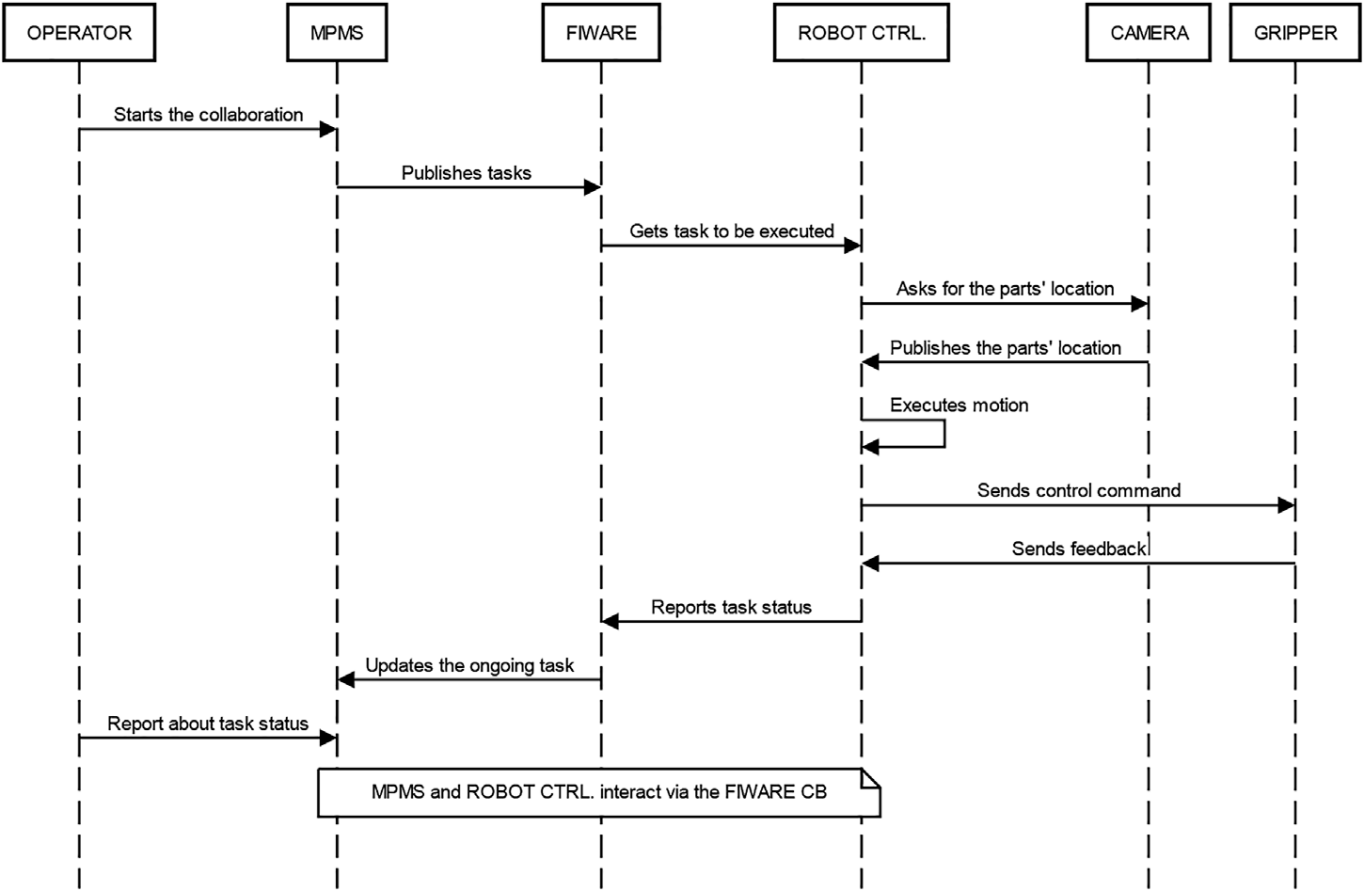
Sequence diagram for the interaction of the different technological components. The user could execute the task through the MPMS interface. Afterward, the MPMS is taking care of publishing and monitoring the tasks in the FIWARE context broker for either the operator or the robot controller (ROBOT CTRL.). The same is done by the robot controller which waits for triggers on when to execute certain actions. This included moving the robot, gathering position data from the camera, and controlling the gripper.

To coordinate the collaboration and consent the transferring of information, the SHOP4CF^6^ architecture was adopted to design and execute the scenario (Zimniewicz, 2020). More precisely, the Task data model^7^ and the Manufacturing Process Management System (MPMS) component (Erasmus et al., 2020) were employed to coordinate the robot controller and the operator. On one hand, the MPMS provided a process modeler to design the process models and a process engine to automatically execute these models. On the other hand, the Tasks were published and monitored in a shared Context Broker (CB), allowing the robot controller to receive triggers on when to perform actions and send to the MPMS Human Machine Interface (HMI) triggers on operator actions. The sequence diagram depicting the interaction among these components is shown in Figure 6. By adopting this architecture, it was possible to integrate operator inputs while ensuring safety and coordination with cyber-physical system components.

### 2.5 Experiment design

To test the hypotheses, an experiment following the SHOP4CF guidelines for user studies was created (Aromaa and Heikkilä, 2020). The study was designed to compare interactions between *a priori* defined parts’ positions and operator-defined parts’ positions. Therefore, a 2 × 2 mixed design with two subsequent balanced randomized user interactions was used. Through this design, two variables were manipulated: the positioning of the parts (user defined vs. *a priori* defined) and the degree of familiarity with the application (first interaction vs. second interaction). To distinguish across the experiments, the following abbreviations are used: *std* for the test with *a priori* part positioning and *usr* for the test with operator-defined part positioning, and Interaction I for the first interaction and Interaction II for the second interaction.

**FIGURE 7:**
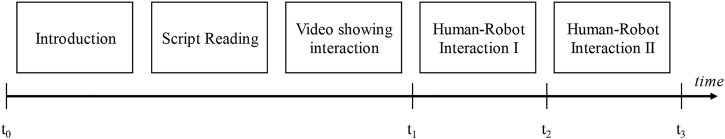
Experiment structure. Between t_0_ and t_1_, the user was presented with the robot cell and the experiment was explained. Afterward, at t_1_, the trust level was measured along with user demographics and informed consent. Between t_1_ and t_2_, the user was then performing the first interaction, which ended at t_2_ with the WDQ. Then, between t_2_ and t_3_, the user was performing the second interaction, which ended at t_3_ with the WDQ.

With this design, the experiment procedure carried out by the authors was as follows; nomenclature and visual representation are shown in Figure 7. Initially, between times t_0_ and t_1_, the users were presented with the robotic cell, and a short explanation of the robot’s safety was given. Afterward, a script describing the human–robot interaction was read, and a video describing the collaboration in the experiment was presented. Then, at time t_1_, informed consent and general user demographics, technology fitness and level of trust in automation (Jian et al., 2000) were collected. Second, between t_1_ and t_2_, Interaction I was performed. During the interaction, the position of the operator was monitored and evaluated as outlined in Section 2.2 for identifying data regarding *H2*. This second part concluded at t_2_ with the user replying to the section of the work design questionnaire (WDQ) (Morgeson and Humphrey, 2006) related to task autonomy for properly identifying data for *H1*. Finally, between t_2_ and t_3_, Interaction II was performed, and similarly to Interaction I, the position of the operator was monitored. The experiment concluded at t_3_ with the user replying to the section of the WDQ related to task autonomy.

## 3 Results

This section reports the results collected during the study. The analysis has been conducted on the collected datasets during the experiments. Some of the datasets are made available in the Supplementary Materials of this article.

### 3.1 User demographics

The user group taking part in the study was composed of 17 individuals, not trained in physical ergonomics, M age = 33.05 years SD = 12.89, M height = 180.11 cm SD = 9.26. Within this group, the technology fitness measured in average hours per week spent with electronic devices was M = 31.15 h, (SD = 8.38), and the group expressed an average trust in automation of M = 4.87 (SD = 1.02) on a scale from 1 to 7. Out of the 17 test subjects, the task was performed correctly by 16 participants. Therefore, one test was discarded.

### 3.2 Task autonomy

**FIGURE 8:**
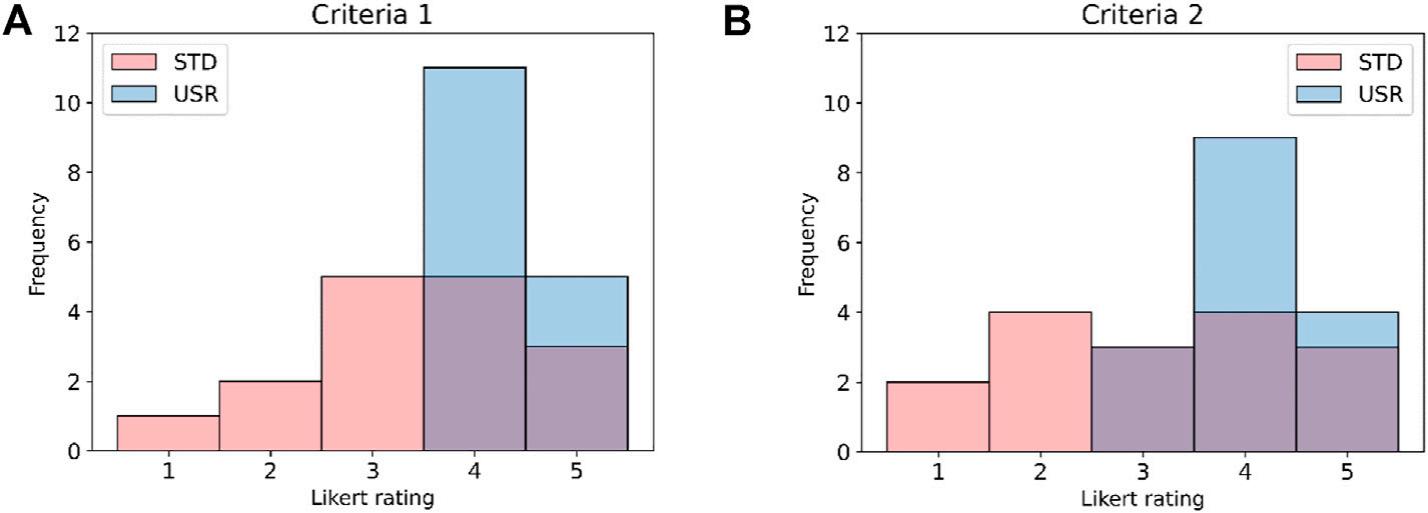
Histogram plots showing the outcomes to the WDQ evaluation collected in the Criteria 1 **(A)** and the Criteria 2 **(B)**. In blue, the USR interaction, and in pink, the STD interaction. The figures show that both criteria received a better rating in the case of the USR interaction.

To measure the task autonomy, the WDQ criteria were as follows: Criteria 1 (“The system gives me a chance to use my personal initiative or judgment in carrying out the work”) and Criteria 2 (“The system provides me with significant autonomy in making decisions”). The responses to these criteria were monitored after each trial, and the results are shown in Figure 8. It is possible to denote that Criteria 1 and Criteria 2 have different ratings; moreover, the *usr* shows a higher rating. Therefore, a Mann–Whitney U test was performed after having identified that the homogeneity of variance assumption for the *t*-test did not hold true, and the Levene test reported *p* < 0.05 (CI = 95%). From these results, it is possible to see that *p* < 0.05 (CI = 95%) for both Criteria 1 and Criteria 2. Therefore, a statistically significant difference among *std* and *usr* responses for the criteria is found, and the *usr* scored better than *std*.

### 3.3 Operators’ posture

**FIGURE 9:**
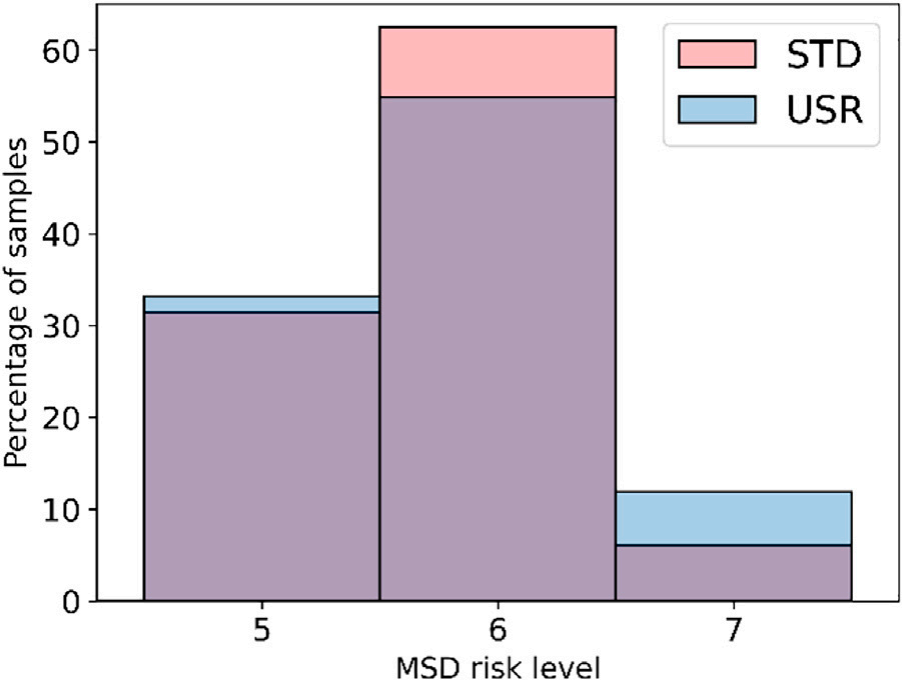
Histogram plot representing MSD risk levels for the two tests calculated through the RULA assessment, the USR in blue and the STD in pink. The figure shows that the STD interaction had a larger number of samples classified with the risk level 6 when compared to the USR interaction. Moreover, the USR interaction reported more samples with risk level 7.

To measure the operators’ posture, the RULA assessment was monitored. As long as the RULA assessment was calculated for each frame in each test, the average MSD risk level was used to analyze the change between *std* and *usr*. The calculated risk levels among the trials are shown in Figure 9. Looking at the figure it is possible to see that there are similar risk levels among *usr* and the *std*. To check any similarity among the two distributions, a Mann–Whitney U test was performed after having identified that the homogeneity of variance assumption for the *t*-test did not hold true, and the Levene test reported *p* < 0.05 (CI = 95%). The outcome of the Mann–Whitney U test was *p* > 0.05 (CI = 95%); therefore, a significant difference between the MSD risk level in *std* and *usr* is not found. Therefore, the hypothesis of statistically significant difference must be rejected, and the samples should be considered to have a similar distribution.

**FIGURE 10:**
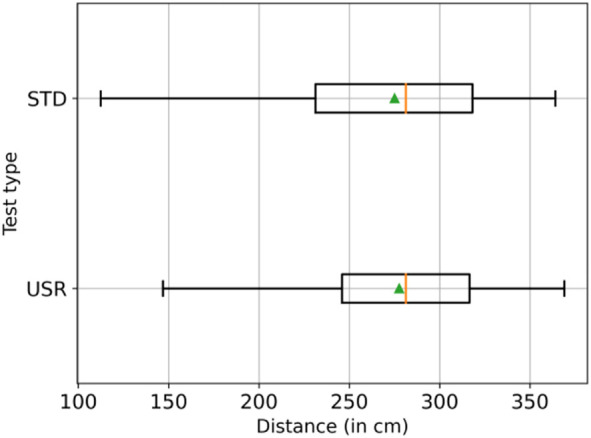
Boxplots representing the distances for the two tests, the means in green and the medians in orange. The means and medians between the two interactions are similar. However, the STD interaction displays a slightly smaller mean, meaning that on average, the users were closer to the robot workspace in the STD interaction.

Furthermore, to measure the usage of the workspace during the experiment, the distance between the robot workspace and operator position was used. As long as the distance was calculated for each frame, the average value was monitored. The measured distance among the trials is shown in Figure 10. Looking at the figure, it is possible to detect similar distances in *usr* and *std*. A Mann–Whitney U test was performed after having identified that the homogeneity of variance assumption for the *t*-test did not hold true, and the Levene test reported *p* < 0.05 (CI = 95%). The outcome of the Mann–Whitney U was *p* < 0.05 (CI = 95%); therefore, a statistically significant difference between the samples is found. Since the distance in *std* M = 275.05 cm, SD = 46.88 and in *usr* M = 277. cm, SD = 44.75, the distance in *usr* is found to be larger than the one in *std*.

### 3.4 Robot performances

**FIGURE 11:**
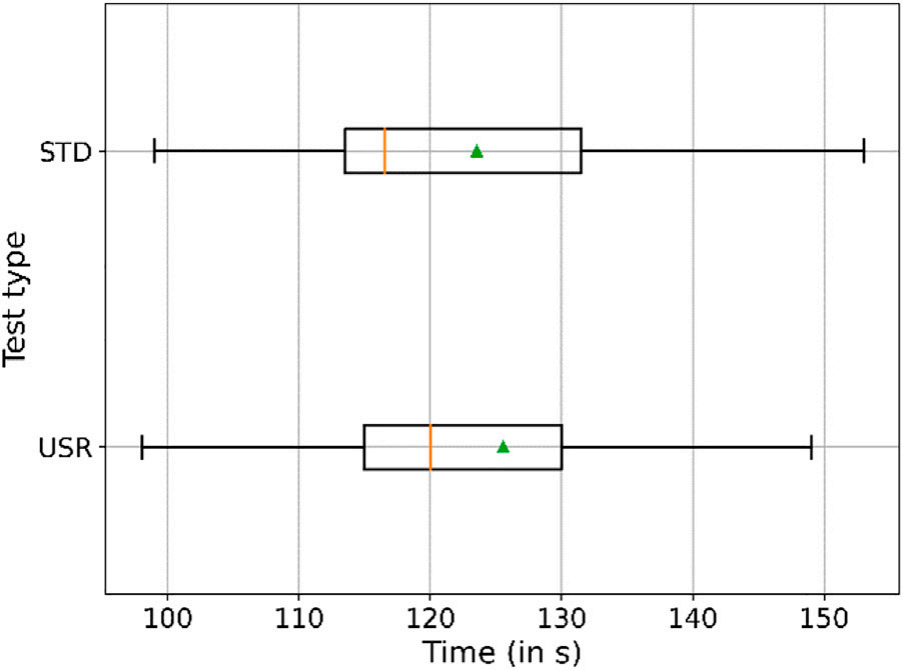
Boxplots representing the time necessary to complete the whole test, the means in green and the medians in orange. The figure displays that the time to complete the activity with STD interaction was shorter than that of USR.

To measure the performance of the task, the time taken for completing the collaboration was monitored. The times taken are shown in Figure 11. The figure shows that *usr* completion times are bigger than *std* completion times. To prove this assumption, a Mann–Whitney U test was performed after having identified that the homogeneity of variance assumption for the *t*-test did not hold true, and the Levene test reported *p* < 0.05 (CI = 95%). The result of the test led to *p* > 0.05 (CI = 95%). Therefore, the hypothesis of statistically significant difference must be rejected, and the samples should be considered to have a similar distribution.

## 4 Discussion

### 4.1 Task autonomy

The responses to the WDQ criteria highlighted that leaving operators to decide where to place the parts can lead to higher task decision autonomy. Therefore, we can conclude that allowing this decision can create a good mental model for task autonomy and *H1* can be accepted. This is aligned with what (Tausch and Kluge, 2020) discovered in their review of performing user tests without real robots. Consequently, we can infer that leaving the decision to the operator on robot tasks is beneficial to the perceived autonomy from the operator.

### 4.2 Posture of the operators during the tests

**FIGURE 12:**
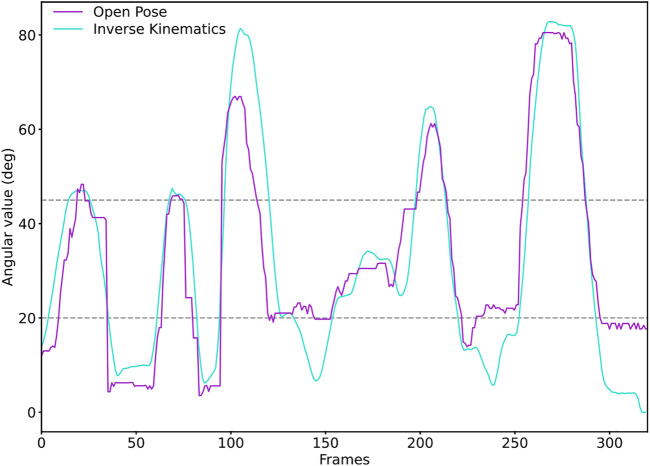
Comparison between the OpenPose angular values and the IK ones for the upper arm. Although differences exist, if the threshold values of the RULA assessment are considered (20° and 45°), the trend between the two evaluations is comparable. Therefore, the final MSD risk level calculated with the two approaches can be considered similar.

The results of the analysis of the operators’ postures show that the level of MSD risk in *usr* is slightly higher, Δ = +0.05, and this is not statistically significant. This outcome is against the hypothesis that higher autonomy should lead to lower level of MSD risk. Therefore, *H2* needs to be rejected. However, no statistical significance was found between the tests, thus suggesting that operators’ decisions did not influence the MSD risk level. Despite this, the risk level measured in both the tests is medium according to the RULA assessment (McAtamney and Nigel Corlett, 1993). To further investigate the results of the RULA ergonomic assessment and identify any shortcomings, an experiment was performed to compare the calculated MSD risk level values with a ground truth. To do so, the angular values of the main arm joints calculated using the method explained in Section 2.2 were compared with the angular values of an arm simulation model developed in Matlab™. To achieve this comparison, a positional tracking experiment was performed. An operator was placed in front of the cameras and, holding the tracker on the hand, performed different arm positions. Afterward, the dataset was used to simulate the arm angular values through an IK algorithm, and the results were compared with the angles calculated using the method outlined in this work. The comparisons are reported in Figure 12. Although differences exist in the values, the trend between the two measurements is similar. Considering both that RULA uses the thresholds of 20° and 45° for the upper arm and that the relative difference between the two tests was measured, we can consider the evaluation through our method good enough for the estimation of the risk levels as also identified by (Kim et al., 2019), thus eliminating the hypothesis of a wrongly performed ergonomic assessment. Consequently, we can conclude that leaving the decision of where to move the parts to the operator does not result in an increased level of MSD risk in situations similar to the experiment conducted.

In addition, the results showed that operators in *usr* kept a larger distance from the robot than those in *std*. Therefore, this suggests that operators learn the robot’s behavior better (the further away, the faster) when they had higher task autonomy. Unfortunately, this did not reflect in different completion times as shown in Section 3.4. Therefore, further studies are necessary to investigate why users did not move farther away from the robot.

### 4.3 Robot performances

The tests showed that *std* led to lower competition times than *usr*. However, this difference was not found to have a strong impact due to the non-statistical significance (*p* > 0.05). Therefore, further tests should be conducted to investigate the matter.

### 4.4 Influences of task familiarity on the interaction

For further investigating if the autonomy had additional implications with other factors in the experiments, a correlation analysis using the Pearson correlation coefficient was conducted on the available measures for the two groups. The results for the group which performed *std* as Interaction I are depicted in Table 2. The results for the group which performed *usr* as Interaction I are depicted in Table 3.

**TABLE 2:** Correlation analysis among the metrics gathered during the experiment for the group which performed the *std* as Interaction I calculated with the Pearson correlation coefficient. The meaningful correlations (*p* < 0.05) are highlighted in bold. The following abbreviations are used: Dist. is used for Distance, Erg. for physical ergonomics, and Aut. for autonomy

		M	SD	Trust scale	Tech. fitness	Dist. *std*	Dist. *usr*	Erg. *std*	Erg. *usr*	Time *std*	Time *usr*	Aut. *std*	Aut. *usr*
1	Trust scale	5.00	1.19	-									
2	Tech. fitness	34.0	6.21	0.50	-								
3	Dist. *std*	295.22	35.49	0.24	0.12	-							
4	Dist. *usr*	276.06	38.05	−0.27	-0.23	0.66	-						
5	Erg. *std*	5.57	0.60	0.04	0.03	-0.22	−0.35	-					
6	Erg. *usr*	5.69	0.54	−0.30	-0.39	0.70	−0.60	0.59	-				
7	Time *std*	129.13	17.63	−0.64	-0.15	−**0.76**	−0.37	-0.16	0.53	-			
8	Time *usr*	123.50	23.29	−0.15	-0.08	-0.68	−**0.80**	0.52	**0.86**	0.59	-		
9	Aut. *std*	2.75	1.06	−0.11	−0.03	−0.11	−0.12	0.49	0.02	-0.17	0.29	-	
10	Aut. *usr*	3.50	0.72	0.21	0.32	0.29	0.01	0.02	−0.36	−0.38	−0.05	0.70	-

**TABLE 3:** Correlation analysis among the metrics gathered during the experiment for the group which performed the *usr* as Interaction I calculated with the Pearson correlation coefficient. The meaningful correlations (*p* < 0.05) are highlighted in bold. The following abbreviations are used: Dist. is used for Distance, Erg. for physical ergonomics, and Aut. for autonomy. For the sake of readability, the *usr* and *std* rows have been swapped compared to Table 2 to show differences between interaction I (*usr*) and interaction II (*std*).

		M	SD	Trust scale	Tech. fitness	Dist. *usr*	Dist. *std*	Erg. *usr*	Erg. *std*	Time *usr*	Time *std*	Aut. *usr*	Aut. *std*
1	Trust scale	4.75	0.88	-									
2	Tech. fitness	28.31	9.67	0.40	-								
3	Dist. *usr*	276.42	50.39	−0.22	0.09	-							
4	Dist. *std*	254.30	47.41	−0.21	0.52	**0.82**	-						
5	Erg. *usr*	5.92	0.70	−0.35	−0.15	−0.04	−0.11	-					
6	Erg. *std*	5.83	0.52	−0.25	−0.26	−0.32	0.06	0.09	-				
7	Time *usr*	127.63	20.80	−0.08	−0.02	−0.31	−0.67	−0.62	0.30	-			
8	Time *std*	118.00	16.10	−0.23	−0.54	−0.64	−**0.78**	0.07	0.23	0.66	-		
9	Aut. *usr*	4.41	0.44	0.02	−0.05	0.37	0.28	−0.22	0.13	−0.21	0.11	-	
10	Aut. *std*	3.91	0.88	−0.08	−0.25	0.12	0.16	−0.05	0.40	0.15	0.13	0.60	-

When looking for similarities across the two groups, the following can be noted. In both cases, the average distance from operator to robot workspace always decreased in the second interaction (see averages of Dist. *std* and Dist. *usr* in Tables 2, 3). Similarly, in both cases, the average time to complete the task decreased in the second interaction (see averages of Time *std* and Time *usr* in Tables 2, 3). This is related to the fact that error-free robot interaction, which, in this case, is always Interaction II, leads to a better acquaintance and familiarity, as also identified by (Miller et al., 2021). Another important factor is the time taken to complete the task. In both scenarios, the time was negatively correlated with the distance kept by the operator when the *std* unfolded. This means that the users correctly learned the robot safety strategy, which adopted slower speeds when they were closer, only when the *std* was active, therefore suggesting a need for the users to interact with a standard configuration at first before allowing the operator to customize its own interaction for learning the safety operation modality. Last, the MSD risk level is always slightly higher in the case of *usr* being active independently on the interaction order, as also shown in Section 3.3.

Then, when looking for differences across the groups, the following can be identified. First, in *usr* as Interaction I (Table 3), the distance kept by the operator in Interaction I (*usr*) is positively correlated with the distance kept in Interaction II (*std*); this indicates that the distance kept in the first interaction has influenced the second interaction. This might be connected to the higher feeling of task autonomy given by the location selection in the first collaboration instance. Second, in *std* as Interaction I (Table 2), the MSD risk level in Interaction II (*usr*) is positively correlated with the time elapsed in the Interaction II (*usr*), meaning the more time, the worse the MSD risk level. Moreover, the distance in the Interaction II (*usr*) is negatively correlated with the time taken. Considering these two points, it is possible to note a connection between the table distance, the time, and the level of MSD risk. This can, on one hand, be connected to the programming of the robot where the closer the users, the slower the robot, as identified in the paragraph before. On the other hand, we see that a closer distance to the table might have led to uncomfortable positions for the users, which prolonged the time in *usr*, thus leading to an overall worse scoring of the physical ergonomics in *usr*. This is similar to what was discovered by (Kar et al., 2015), where faster movements led to lower operator risk. However, this held true in just one scenario, and the overall analysis yielded that operators in *usr* were, on average, farther away, as shown in Section 3.3. Therefore, further tests will be necessary to investigate this last finding, also considering that no statistical significance was found on the difference of competition times between *std* and *usr*.

### 4.5 Study limitations

During the study, some limitations were observed. In this section, the two main drawbacks are explained.

The experiment script read to the test participants contained a detailed step-by-step description of the experiment in the following order. First, how *std* worked. Second, how *usr* worked and how the parts’ positioning might influence the user. Finally, how the robot was programmed (i.e., the closer, the slower). Therefore, no other goals other than completing the activity were assigned to users. During the tests, this resulted in a high variability on the part displacement. Some users placed the parts closer to the robot and some users placed them farther away from the robot. The authors always asked in an open-ended question why that was the case and some of the answers were as follows: “I placed the part closer to the robot so I can be faster although this leads to a bad position for me” or “I placed the parts closer to me so I can handle them better” or “I like the parts in the center of the table, and they are easy to handle.” Considering these observations, it is possible to denote that not having a clear objective on what to optimize for (i.e., robot speed, safety, or physical ergonomics) was having some degree of impact. Therefore, users had the choice to select randomly what to optimize for, and this was probably influenced by different individuals’ backgrounds, as already identified by (Miller et al., 2021). This might have led to the unexpected results observed in this study like the increase in the time necessary to complete the task with *usr*. Therefore, the results of this study should be considered only in situations where the users are requested to accomplish a pick-and-place task without any clear objective on what to focus apart from completing the activity.

Aside from this, it is important to underline that the employed methodology for the calculation of the MSD risk levels was the RULA assessment, through a software pipeline which analyzed images. The software pipeline has been developed by the authors and despite proving to be accurate, as described in Section 4.2, some limitations might still be present due to the CV approach based on OpenPose. Other literature using the same approach as the study by (Kim et al., 2021) reported that the approach could be affected by the placement and resolution of the cameras. Despite this, considering that the same software pipeline has been used both for *usr* and *std*, the delta differences in the physical ergonomics between the two groups should still be valid.

## 5 Conclusion

In this work, a study to investigate the influence of task autonomy on operator physical ergonomics and robot performances in industrial human–robot collaboration *via* a user study has been presented. The results yielded that higher task autonomy can be achieved by letting an operator decide the position of handled parts, and this does not lead to statistically significant differences in the overall task efficiency, nor an increase in the MSD risk level. However, this result might have been influenced by two drawbacks in the experiment design. First, it was observed that a clear objective for the task was not communicated to the participants. Therefore, users might have optimized for different aspects (e.g., posture or speed), leading to the observed results. Second, the estimation of the MSD risk level based on RULA might have been affected by the camera resolution of the camera displacements as identified by previous literature. Therefore, the measured risk level might not have been correct. Despite these limitations, this study highlighted that robotic systems able to let the operators decide about some task parameters like the parts’ positioning can be beneficial and that the SHOP4CF architecture allows us to integrate such scenarios. However, proper consideration should be taken to understand how users decide for certain application aspects, and further research is needed to ensure user wellbeing on this aspect. Therefore, with the published open-source software and dataset for the physical ergonomics, the authors would like to encourage other researchers to further study on the topic.

## Data Availability

The original contributions presented in the study are included in the article/Supplementary Material; further inquiries can be directed to the corresponding author.

## Footnotes

1
http://www.fcc.chalmers.se/software/ips/ips-imma/

2
https://www.plm.automation.siemens.com/global/en/products/manufacturing-planning/human-factors-ergonomics.html

3
https://ergo-plus.com/wp-content/uploads/RULA.pdf

4
https://github.com/matteopantano/human-ergonomic-module

5
https://ros-planning.github.io/moveit_tutorials/doc/hand_eye_calibration/hand_eye_calibration_tutorial.html

6
https://shop4cf.eu/

7
https://github.com/SHOP4CF/data-models
